# Antimicrobial, modulatory and chemical analysis of the oil of *Croton limae*

**DOI:** 10.1080/13880209.2017.1355926

**Published:** 2017-07-25

**Authors:** Tiago Rodrigues Leite, Maria Arlene Pessoa da Silva, Antônio Carlito Bezerra dos Santos, Henrique Douglas Melo Coutinho, Antonia Eliene Duarte, José Galberto Martins da Costa

**Affiliations:** a Post-graduate Program in Molecular Bioprospection, Cariri Regional University, Crato, Brazil;; b Department of Biological Sciences, Regional University of Cariri, Crato, Brazil

**Keywords:** Marmeleiro-prateado, *Staphylococcus aureus*, *Escherichia coli*; *Pseudomonas aeruginosa*, *Klebsiella pneumonia*, *Candida tropicalis*, *Candida krusei*, *Candida albicans*, essential leaf oil, chemical constituents

## Abstract

**Context:***Croton* sp. are plants with a well-reported antimicrobial activity*. Croton limae* A.P. Gomes, M.F. Sales P.E. Berry (Euphorbiaceae), known as ‘marmeleiro-prateado’, is commonly used to manage abdominal pain in Brazil.

**Objective:** This work evaluates the phytochemical composition, antimicrobial and modulatory activities of the essential oil of *C. limae* leaves (EOCL).

**Materials and methods:** The minimum inhibitory concentration (MIC) and the modulation of the antibiotic activity were determined using a microdilution method. The concentration of EOCL ranged between 512 and 8** **μg/mL. *Staphylococcus aureus*, *Escherichia coli, Pseudomonas aeruginosa*, *Klebsiella pneumonia*, *Candida tropicalis*, *C. krusei* and *C. albicans* strains were used in the MIC and modulation assays. The antibiotics, amikacin, gentamicin and neomycin, and the antifungals, amphotericin B, benzoylmetronidazole and nystatin, were used in concentrations ranging between 2500 and 2.5** **μg/mL. The phytochemical analysis of the EOCL was performed through gas chromatography coupled to a mass spectrometer (GC/MS).

**Results:** Only *Staphylococcus aureus* was inhibited by a clinically relevant concentration of EOCL (MIC 512 μg/mL). Synergism between the EOCL and amikacin against *S. aureus* (9.76 μg/mL) and *E. coli* (39.062 μg/mL); neomycin against *E. coli* (2.44 μg/mL); and benzoylmetronidazole against *C. krusei* (256 μg/mL) were observed. The GC/MS analysis identified cedrol, eucalyptol and α-pinene as the main compounds of EOCL.

**Conclusion:** EOCL inhibited the growth of *S. aureus* and potentiated the antibiotic and antifungal effects of drugs against all bacterial and *Candida* strains, respectively.

## Introduction

The intensive use of antibiotics and fungicides causes microorganisms to develop defenses which then result in resistance to drugs, thus limiting the treatment options against infections (Varaldo 2002). The swift evolution of resistance to antimicrobials and the lack of discovery of new drugs have motivated the search for alternatives, such as multidrug treatment approaches (Keith et al. 2005). Studies from various authors have shown that species of the *Croton* genus have antimicrobial activity against diverse species of bacteria and fungi, in addition to a modulatory activity of antibacterial and antifungal drugs (Costa et al. 2008; López 2010; Nader 2010).

*Croton limae* A.P. Gomes, M.F. Sales P.E. Berry (Euphorbiaceae), commonly referred to as ‘marmeleiro-prateado’, is found between arboreal and shrubby habitats; it has a silver to orange-yellow, dentate-lepidote to lepidote surface. It displays an alternate phyllotaxis; the leaf is simple, oblong-elliptic to oblong and membranaceous with brochidodromous venation. The flowers are unisexual, forming inflorescence of terminal and subterminal raceme type, with one to three bracts per flower. The staminate flowers have five free sepals and 10–15 stamens. The pistillate ones are sessile or subsessile with five sepals. Petals are generally absent or rudimentary and pistils are multifid. The fruits are globular capsules, the surface orange-yellow, with persistent sepals (Gomes et al. 2010). According to Souza et al. (2014), *C. limae* is used in traditional medicine to treat stomachache.

*C. limae* is a species found in areas of Cerrado in the Chapada do Araripe, in the south of Ceará, and was identified in 2009. However, there is no literature referring to the antimicrobial activity of this species. Therefore, the objective of this study is to evaluate the antimicrobial and modulatory activity of *C. limae*.

## Material and methods

### Collection and identification of botanical material

The *C. limae* plant material was collected in January 2010 (8:00 a.m.), from a Brazilian savannah biome named ‘Cerrado’, at the Chapada do Araripe (Araripe Plateau), in the Barreiro Novo County (07° 17.77′ S and 39° 32.62′ W, at 923 m height). Fresh leaves were collected for essential oil extraction and branches with flowers were used for botanical identification. The collection of plant material was performed by Mr. Tiago Rodrigues Leite.

The identification of the botanical material was carried out by the taxonomist Dr. Margareth Ferreira de Sales at the Herbarium Sérgio Tavares (HST) of the Federal Rural University of Pernambuco (UFRPE), Recife – PE. The voucher specimens were kept or stored at the Herbarium Caririense Dárdano de Andrade-Lima (HCDAL) of the Cariri Regional University, under number 6285.

### Extraction of the essential oil of *Croton limae*

The essential oil of *C. limae* fresh leaves was extracted using the hydrodistillation technique. Triturated fresh leaves (480.52 g) were immersed in a flask containing a quantity of distilled water sufficient to entirely cover them and then coupled to a hydrodistillation unit. Two hours after the start of boiling, the essential oil was separated by adding sodium sulfate for the complete removal of water. Next, the mixture was kept refrigerated for 24 h and taken out of the flask with the aid of a glass pipette with cotton wool on the end (to avoid taking out the sodium sulfate), then stored in a labeled flask in the refrigerator at 8–12 °C until used.

### Analysis of the chemical composition of the *Croton limae* essential oil

The analysis of the chemical composition of the essential oil was performed at the Federal University of Piauí, using a gas chromatography coupled to mass spectrometer (GC/MS) in a Shimadzu instrument with mass selective detector QP5050A system, operating at 70 eV of ionization energy. Capillary column Agilent DB-5HT was used (30 m × 0.25 mm of internal diameter) with the following specifications: gun temperature of 270 °C and detector temperature of 290 °C, using helium gas as carrier gas (1.0 mL/min); linear speed of 47.3 cm/sec; total flux of 24 mL/min; carrier flux of 24 mL/min; pressure of 107.8 kPa; the heating temperature of the column was set to 60 °C (2 min) to 180 °C (1 min) at 4 °C/min and 180-260 °C at 10 °C/min (10 min). The identification of the compounds was carried out by comparisons between the respective mass spectrometer and patterns registered in the Wiley 229 database and between the retention index calculations and specialized literature values (Adams 2001).

### Evaluation of the antimicrobial activity of *Croton limae*

The biological tests were performed in the Laboratory of Microbiology and Molecular Biology of the Regional University of the Cariri (URCA), Crato, CE. The essential oil solution (220 mg/mL) was prepared as a stock solution, dissolved in 1 mL of dimethyl sulfoxide (DMSO; Merck, Darmstadt, Germany). Next, the solution was diluted with distilled water, reaching a concentration of 1024 μg/mL.

The microorganisms used in these assays were obtained from the National Institute of Health Quality Control (INCQS) of the Oswaldo Cruz Foundation. Four standard strains (a Gram-positive *Staphylococcus aureus* ATCC 25923 and three Gram-negative ones: *Escherichia coli* ATCC 10536, *Pseudomonas aeruginosa* ATCC 15442 and *Klebsiella pneumoniae* ATCC 4362) were used. Two other multidrug resistant strains (MDR), *Escherichia coli* EC 27 and *Staphylococcus aureus* SA 358, obtained from the Federal University of Paraiba (UFPB), Brazil, were also used. Three fungal strains from the *Candida* genus were assayed: *Candida tropicalis* ATCC 40042, *C*. *krusei* ATCC 2538 and *C. albicans* ATCC 40006. All the strains were inoculated into brain–heart infusion (BHI) culture medium at 3.8% and incubated for 24 h at 35 °C with forced air circulation in order to be reactivated. In the preparation of the inoculum, 100 μL of the strain was added in 1 mL of 10% BHI and then incubated at 37 °C for 24 h.

### Minimum inhibitory concentration (MIC)

The broth microdilution method was used to determine the MIC of the essential oil in 96-well microdilution plates. 1:1 serial dilutions were carried out with the plates in the numerical position (horizontal), adding 100 μL of the culture medium with the strains (inoculum and BHI 10%) and 100 μL of the essential oil in each well. Only the inoculum was placed in the final well, as this acted as the control. The concentrations varied from 512 to 8 μg/mL for each microorganism tested.

The material was incubated in a microbiological incubator at a constant temperature of 35 ± 2 °C for 24 h (Javadpour et al. 1996). After incubation, 20 μL of Resazurin sodium red dye (Sigma-Aldrich, St. Louis, MO) was added to each well in the antibacterial tests, letting it rest for 1 h at room temperature. Regarding the interpretation of the results, the wells which stayed blue were considered a positive result and those which went red were considered negative (Salvat et al. 2001). The interpretation of the antifungal activity was carried out through observation of turbidity. All assays were performed at least 3 times and the most common result was used.

### Modulatory activity assays

For the evaluation of the essential oil as a modulator of antibiotic and antifungal activity, the MIC of the standard antibiotics and fungicides was determined in the presence and absence of the oil using the microdilution method at subinhibitory concentrations (MIC 1/8). The antibiotics, amikacin, gentamicin and neomycin, and the antifungals, amphotericin B, benzoylmetronidazole and nystatin, were used. Benzoylmetronidazole was used as a fungicide in this study due to recent findings of its activity against *Candida* spp. (Santos et al. 2012, 2013; Souza et al. 2012). Two strains of multiresistant bacteria were used, *Staphylococcus aureus* SA 358 and *Escherichia coli* EC 27, and the three previously named strains of fungi.

The antibiotic and fungicides solutions were prepared with distilled water at a concentration of 5000 μg/mL. A volume of 100 μL of each solution was serially diluted (1:1) in the wells containing the BHI broth at 10% and the suspension of the microorganism (1:10) in microdilution plates in the alphabetical position (vertical). The concentrations of the drugs in the culture medium varied from 2500 to 2.5 μg/mL. The incubation of the assays and the interpretation of results are the same as for those previously referred to for the MIC. The control consisted of a serial dilution of the antibiotic in the culture medium made up of 1350 μg of BHI and 150 μL of inoculum. All assays were performed at least 3 times and the most common result was used.

## Results

The yield of the essential oil from the *Croton limae* fresh leaves, calculated from their mass, was 0.36%. In the chemical analysis of the oil, 14 compounds were identified and quantified, representing 84.7% of the total chemical composition. The main compounds were cedrol (28.4%), eucalyptol (17.4%) and α-pinene (13.8%). This corresponds to 59.6% of the total composition of the oil. The chemical compounds identified and their respective quantities, retention time and Kovats index (Adams 2001), are summarized in Table 1.

**Table 1.** Chemical constituents of the essential oil of *C. limae* leaves.

Constituents	RT (min)	RI	(%)
α-Pinene	5.2	939	13.8
β-Pinene	6.2	980	3.0
β-Myrcene	6.8	991	1.5
*p*-Cymene	7.7	1026	4.2
Eucalyptol	7.8	1033	17.4
Linalool	10.1	1098	2.5
Cryptone	12.8	1186	1.3
*p*-Cymen-8-ol	12.8	1189	1.3
β-Caryophyllene	20.1	1418	3.8
α-Humulene	21.1	1452	2.3
Alloaromadendrene	21.2	1458	1.2
Spathulenol	22.9	1576	2.8
Caryophyllene oxide	23.7	1581	1.2
Cedrol	24.9	1589	28.4
Total identified			84.7

RT: retention time; RI: Kovats index (Adams 2001).

In the analysis of the results obtained for the antibacterial activity of the essential oil against the Gram-positive and Gram-negative standard strains, it was noted that the minimum inhibitory concentration (MIC) against the *S. aureus* strain was equal to 512 μg/mL. However, the MIC against *E. coli*, *P. aeruginosa* and *Klebsiella pneumoniae* was equal or superior to 1024 μg/mL; thus, there was no significant antibacterial activity from a clinical perspective (Table 2).

**Table 2.** MIC of the essential oil of *C. limae* against the standard bacterial strains.

Bacteria	MIC (μg/mL)
*Escherichia coli* ATCC 10536	≥1024
*Klebsiella pneumoniae* ATCC 4362	≥1024
*Pseudomonas aeruginosa* ATCC 15442	≥1024
*Staphylococcus aureus* ATCC 25923	512

When the essential oil of *C. limae* was associated with the antibiotic amikacin, it enhanced its activity against *S. aureus*, reducing the MIC from 19.53 to 9.76 μg/mL and against *E. coli*, it reduced the MIC from 312.5 to 39.062 μg/mL. It also enhanced the activity of the antibiotic neomycin, reducing its MIC from 78.125 to 2.44 μg/mL against the *E. coli* strain (Table 3).

**Table 3.** Modulatory activity of the essential oil of *C. limae* against the multiresistant bacterial strains.

	*Staphylococcus aureus*SA 358		*Escherichia coli* EC 27	
Antibiotic	Control	EOCL (μg/mL)	Control	EOCL (μg/mL)
Amikacin	19.53	9.76	312.5	39.062
Gentamicin	2.44	2.44	39.062	39.062
Neomycin	9.76	9.76	78.125	2.44

EOCL: essential oil of *Croton limae*.

The oil exhibited a MIC ≥1024 μg/mL for the three species of *Candida* tested, which is not clinically significant (Table 4). However, when associated with the fungicide benzoylmetronidazole, it showed modulatory activity against two fungal strains; for *C. krusei*, the effect was synergistic, reducing the MIC from 1024 to 256 μg/mL; and for *C. albicans,* it was antagonistic, increasing the MIC from 128 to 1024 μg/mL (Table 5).

**Table 4.** MIC of the essential oil of *C. limae* against the standard fungi strains.

Fungi	MIC (μg/mL)
*Candida albicans* ATCC 40006	≥1024
*Candida krusei* ATCC 2538	≥1024
*Candida tropicalis* ATCC 40042	≥1024

**Table 5.** Modulatory activity of the essential oil of *C. limae* against fungal strains.

	*C. albicans* ATCC 40006		*C. krusei* ATCC 2538		*C. tropicalis* ATCC 40042	
Fungicides	Control	EOCL (μg/mL)	Control	EOCL (μg/mL)	Control	EOCL (μg/mL)
Amphotericin B	1024	1024	1024	1024	1024	1024
Benzoylmetronidazole	128	1024	1024	256	1024	1024
Nystatin	1024	1024	1024	1024	1024	1024

EOCL: essential oil of *Croton limae.*

## Discussion

In the analysis of the chemical composition of *Croton blanchetianus* Baill., Angélico (2011) noted the presence of 15 chemical compounds, with the majoritarian compounds being cedrol (28.4%), eucalyptol (17.4%) and α-pinene (10.5%); thus, their results corroborate with those of this study. Souza et al. (2006) analyzed the chemical composition of the fixed oil from the bark of *Croton cajucara* Benth. from the regional ‘Ver-o-Peso’ market, located in Belém, Pará, and noted the occurrence of alloaromadendrene, linalool and spathulenol in its composition; these compounds were also detected in our study.

The only result indicating a clinically relevant antibacterial concentration was observed against *Staphylococcus aureus*, with a MIC of 512 μg/mL. According to Houghton et al. (2007), any natural product with a biological effect demonstrated with a concentration higher than 1 mg/mL cannot be considered clinically relevant due to the impossibility to be dispersed in the plasma volume. A similar result was found by Azevedo (2010), where the essential oil of *C. cajucara* (red variety) showed antibacterial activity against *S. aureus*.

According to Oliveira et al. (2006), the interference that the essential oil can cause with the action of the antibiotics generally varies in accordance with the type of antibiotic, the type of associated essential oil tested and the type of bacterial strain. In testing the modulatory activity of the essential oil of *C. blanchetianus*, Angélico (2011) observed that there was a potentiation of the action of the aminoglycoside antibiotics, kanamycin, amikacin and gentamicin, in the interaction with the *Bacillus cereus* strain. Nonetheless, he did not observe any interference with the activity of the antibiotics in contact with the *S. aureus* strain, thus differing from the results found in this study for the same bacterial strain. Canton and Onofre (2010) observed in their experiments that the extracts and essential oil of *Baccharis dracunculifolia* DC (Asteraceae) against *E. coli* β-lactamase negative and *S. aureus* can interfere synergistically or antagonistically with the action of antibiotics when associated with them.

Nader (2010), while analyzing the antimicrobial potential of vegetable extracts from a Cerrado area against *S. aureus* strains, also noted antibacterial activity of *Croton* species against this strain; the chloroform and hexane extracts of *Croton antisyphiliticus* Mart. showed bactericidal activity and significant antimicrobial activity, affecting five out of the seven strains tested, with the MIC varying between 0.12 and 0.50 mg/mL. In a further test, the chloroform extract of *C. antisyphiliticus* considerably affected all 20 tested strains; for 17 of them, the MIC was 1.03 mg/mL and for the remaining ones, it was 4.15 mg/mL.

The essential oil of *Croton zehntneri* Pax et Hoffm. inhibited the growth of *Shigella flexneri*, *S. aureus, E. coli* and *Streptococcus* β*-haemolyticus* in the susceptibility test of antibacterial activity. Regarding the MIC, the oil only demonstrated significant activity for *S. flexneri* at concentrations of 1000, 500, 100 and 50 μg/mL, with a MIC equal to 50 μg/mL (Costa et al. 2008).

In this study, the synergistic activity seen in the *C. krusei* strain and the antagonistic one in the *C. albicans* strain suggest that differences in the adaptive characteristics between the two-tested species in some way contribute to or impair the activity of the *C. limae* essential oil when associated with the fungicide benzoylmetronidazole. According to Granowitz and Brown (2008), the antagonism resulting from the combination of antibiotics could be attributed to mutual chelation.

While working with the essential oil of *C. cajucara*, the red variety, and corroborating with the result found for *C. albicans* in this study, Azevedo (2010) verified the absence of antifungal activity against *C. albicans*. Likewise, López (2010) did not find antifungal activity against *C. albicans* when analyzing the brute methanol extract of the leaves and bark of *Croton urucurana* Baill. by using the agar well method.

Different results were found by Fontenelle et al. (2008) in their research on the antifungal activity of the essential oils *Croton argyrophylloides* Muell., *Croton nepetaefolius* Baill. and *C. zehntneri* against *C. albicans*, *C. tropicalis* and *Microsporum canis*, using the diffusion in gel well method and the broth microdilution method for the MIC. These authors observed that *C. nepetaefolius* and *C. argyrophylloides* only demonstrated activity against *M. canis*, while the essential oil of *C. zehntneri* was effective against all the fungi tested.

According to Chang et al. (2003), the cedrol extracted from the heartwood of the species *Taiwania cryptomerioides* Hayata (Taxodiaceae) has antifungal activity against *Coriolus versicolor* and *Laetiporus sulphureus*. As stated by Dorman and Deans (2000), α-pinene, β-pinene and linalool have antibacterial activity against diverse bacterial species, including those of this study. However, α-pinene and linalool do not show activity against *P. aeruginosa.* α-Pinene and β-pinene were found to inhibit the growth of the Gram-positive bacteria, *S. aureus, Staphylococcus epidermis* and *Bacillus subtilis*; the Gram-negative bacteria, *E. coli, Salmonella typhi* and *P. aeruginosa*; and the fungi *C. albicans* and *Candida kefyr* (Ghasemi et al. 2005).

According to Siti Humeirah et al. (2010), α-pinene showed a growth inhibitory effect, which varied from moderate to strong, against the bacterial and fungal species *S. epidermidis*, *C. albicans*, *Trichophyton rubrum* and *M. canis, S. aureus, E. coli.* Meanwhile linalool showed a lower index of microbial inhibition. Leite et al. (2007) perceived in their research that α-pinene and β-pinene have large antimicrobial potential against *S. aureus*, *S. epidermidis*, *Streptococcus pyogenes* and *Streptococcus pneumoniae*.

According to Mahboubi and Kazempour (2009), α-pinene shows activity against *S. aureus*, *B. cereus* and the fungi *Aspergillus niger* and *C. albicans.* Hence, the presence of these compounds in the essential oil of *C. limae* is responsible for the antibacterial activity verified in this study.

Phytochemicals are small, organic biomolecules, which are generally hydrophobic and designated as naturally occurring antibiotics. Cytoplasm membrane coagulation, proton pump and electron flux damage, and active transport imbalances are some of the mechanisms of actions responsible for providing the antimicrobial property of phytochemicals (Sikkema et al. 1995; Carson et al. 2002). It is believed that these biological events do not occur separately, as some are activated as a consequence of others (Sikkema et al. 1995; Burt 2004).

The outer membrane of Gram-negative bacteria is made up of lipopolysaccharide. This membrane forms a hydrophobic barrier which restricts the diffusion of hydrophobic compounds through this coating (Nikaido and Vaara 1985). It is probably for this reason that the *C. limae* oil did not inhibit the growth of *E.* coli, *P. aeruginosa* and *K. pneumoniae*, which are Gram-negative bacteria.

Andrews et al. (1980) noted in studies that α-pinene destroys the cellular integrity of Gram-positive bacteria and inhibits the respiratory chain enzymes. Besides this activity, Gram-negative bacteria were weakly affected by this compound, possibly due to their concentration in the essential oil.

In their research, Singh et al. (2006) observed that α-pinene increased the conductivity levels in the membrane, indicating that it caused stress which resulted in the rupture of the membrane integrity. Harrewijn et al. (2001) have suggested that membrane rupture is one of the mechanisms responsible for the antifungal and antibacterial activity of monoterpenoids.

In light of the results shown for cedrol, eucalyptol, α-pinene, β-pinene and linalool in various studies, there exists the possibility that these substances, in isolation or in combination, are responsible for the antibacterial activity and modulatory activity of the antibacterial and antifungal drugs demonstrated by the essential oil of the fresh leaves of *C. limae*. We suggest that these substances act by causing a rupture in the plasma membrane, affecting the proton pump and causing a damage on the electron flux, and on active transport imbalance through the membrane, in addition to inhibiting respiration in the mitochondria of the Gram-positive bacterium *S. aureus* and the fungal strains.

## Conclusions

*Staphylococcus aureus* is sensitive to the essential oil from the fresh leaves of *Croton limae*, with a MIC equal to 512** **μg/mL. The essential oil of *C. limae* demonstrated its ability to potentiate the action of the antibacterial aminoglycoside drugs amikacin, in *S. aureus* and *Escherichia coli* strains, and neomycin, in *E. coli* strains. In the modulatory test, the *C. limae* essential oil showed the best result when associated with amikacin against *E. coli* strains. When associated with the fungicide benzoylmetronidazole, the *C. limae* essential oil showed synergistic activity in *Candida krusei* cultures and antagonistic activity in *Candida albicans* cultures.
